# Aberrant DNA methylation profile exacerbates inflammation and neurodegeneration in multiple sclerosis patients

**DOI:** 10.1186/s12974-019-1667-1

**Published:** 2020-01-14

**Authors:** Naiara Celarain, Jordi Tomas-Roig

**Affiliations:** Girona Neuroimmunology and Multiple Sclerosis Unit (UNIEM), Dr. Josep Trueta University Hospital and Girona Biomedical Research Institute (IDIBGI), Girona, Spain

**Keywords:** Multiple sclerosis, DNA methylation, Environmental risk factors, Inflammation, Neurodegeneration

## Abstract

Multiple sclerosis (MS) is an autoimmune and demyelinating disease of the central nervous system characterised by incoordination, sensory loss, weakness, changes in bladder capacity and bowel function, fatigue and cognitive impairment, creating a significant socioeconomic burden. The pathogenesis of MS involves both genetic susceptibility and exposure to distinct environmental risk factors. The gene x environment interaction is regulated by epigenetic mechanisms. Epigenetics refers to a complex system that modifies gene expression without altering the DNA sequence. The most studied epigenetic mechanism is DNA methylation. This epigenetic mark participates in distinct MS pathophysiological processes, including blood–brain barrier breakdown, inflammatory response, demyelination, remyelination failure and neurodegeneration. In this study, we also accurately summarised a list of environmental factors involved in the MS pathogenesis and its clinical course. A literature search was conducted using MEDLINE through PubMED and Scopus. In conclusion, an exhaustive study of DNA methylation might contribute towards new pharmacological interventions in MS by use of epigenetic drugs.

## Background

**Fig. 1:**
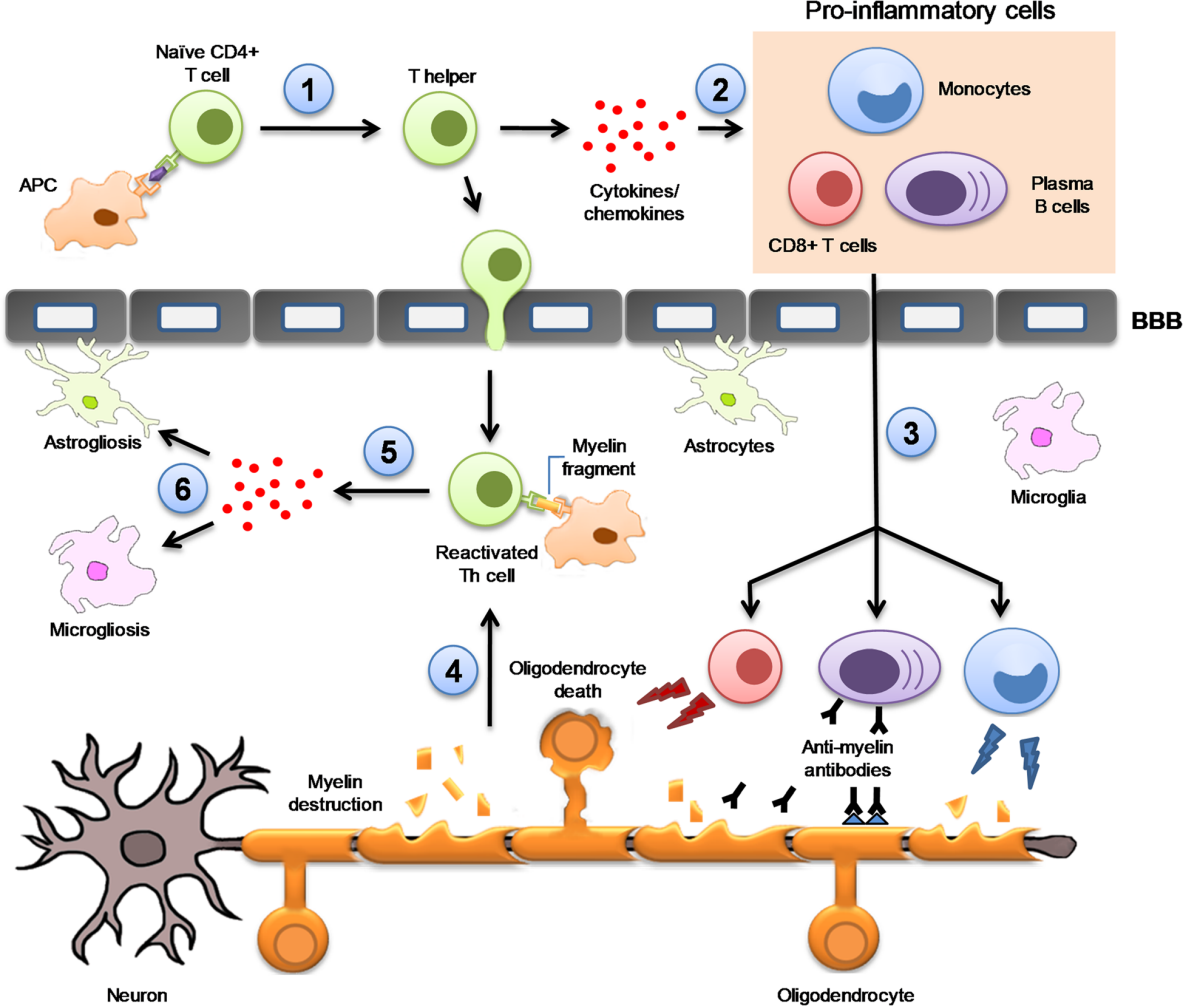
The underlying pathophysiological mechanism of MS. In the first instance, autoreactive CD4+ T cells are activated in the periphery by antigen presenting cells (APC) that present, in conjunction with class II MHC molecules, similar antigens to those synthesised by the CNS. (1) This interaction activates the differentiation of CD4+ T naïve cells into CD4+ T helper cells (Th). (2) Upon activation, Th produces interferon-gamma (IFN-γ), a cytokine responsible for recruiting CD8+ T cells, B cells and monocytes in the periphery. (3) These proinflammatory cells migrate to the blood–brain barrier (BBB) and pass into the CNS. Inside the brain, plasma B cells produce auto-antibodies against CNS self-antigens contributing to myelin sheath damage. This process is aggravated when infiltrated cytotoxic CD8+ T cells attack oligodendrocytes causing their destruction and neuronal death. Monocytes, on the other hand, increase local inflammatory response by releasing proinflammatory cytokines and contributing to demyelination through myelin phagocytosis. (4) In parallel, infiltrated CD4+ T cells are reactivated upon interaction with myelin fragments presented by resident APCs which favours (5) proinflammatory cytokines and chemokines release, (6) astrogliosis and microgliosis

Multiple sclerosis (MS) is an autoimmune, inflammatory, demyelinating and neurodegenerative disease of the central nervous system (CNS) [1]. As a result of myelin sheath destruction, the electric impulse between neurons is inefficient, and thus the initial symptoms appear [2]. Although symptoms differ from each MS patient, the most common ones include incoordination, sensory loss, weakness, changes in bladder capacity and bowel function, fatigue, and cognitive impairment [3]. Therefore, MS has serious negative effects on the health-, social, and work-related issues of patients and their families, creating a significant socioeconomic burden [4]. The aetiology of MS is still unknown and requires a close interaction between genetic susceptibility and exposure to environmental agents. Synergistic effect of these risk factors would be responsible for triggering autoimmunity in MS patients. For this reason, the underlying mechanisms involved in the MS pathogenesis can differ among patients. Common mechanisms are observed in the pathophysiology of disease and listed next. Autoreactive CD4+ T cells are activated in the periphery [1] by antigen-presenting cells (APC), that present via the class II major histocompatibility complex (MHC) receptor an amino acid similar to myelin peptides synthesised in the CNS. This interaction activates the differentiation of the CD4+ T naïve cells into CD4+ T helper cells [5]. Upon activation, the Th1 subtype produces interferon gamma (IFN-γ) [6], a cytokine responsible for recruiting CD8+ T cells, B cells and monocytes in the periphery [7]. Then, these proinflammatory cells migrate to the blood–brain barrier (BBB) throughout the bloodstream, where they can adhere to the BBB endothelium [8]. In a healthy brain, immune cells are circulating freely in the meninges orchestrating immune surveillance of the CNS [9]. In MS, the BBB displays an aberrant expression and organisation of the endothelial tight junctions [10] that favours massive lymphocyte trafficking into the brain [11]. Infiltrated CD4+ T cells in the CNS are reactivated upon interaction with the resident APCs [12]. Afterwards, the reactivated CD4+ T cells release a variety of proinflammatory cytokines and chemokines [13], resulting in astrogliosis [14] and microgliosis [15]. This process is exacerbated when infiltrated cytotoxic CD8+ T cells attack oligodendrocytes, causing their destruction and neuronal death [16]. In parallel, plasma B cells produce antibodies against CNS self-antigens, contributing to myelin sheath damage [17]. Plasma B cells in coordination with monocytes increase the local inflammatory response by reactivating the autoreactive CD4+ T cells [18] (Fig. 1). T cell–mediated axonal injury contributes to trophic/metabolic support deficiency from oligodendrocytes as well as a lack of energy by releasing soluble inflammatory molecules [19]. The pathophysiology of MS suggests a complex interaction between the genetic and environmental risk factors [20] regulated by epigenetic mechanisms. Epigenetics can provide a stable heritable base for understanding the underlying mechanisms involved in MS [21].

## Genetic, epigenetic and environmental factors

The robust susceptibility loci that confer risk for MS are the human leukocyte antigen (HLA) system, which is located in the short arm of chromosome 6 [22]. However, only 27% of MS heritability can be explained by the genetic variants of the HLA system [23], which supports a prominent contribution of the environment to the MS pathogenesis. Indeed, Epstein Barr virus (EBV) infection, tobacco smoking, vitamin D deficiency, diet style and sun light exposure are critically involved in MS susceptibility [24, 25]. The individual genetic background in combination with the environmental risk factors increase the probability of developing MS. Epigenetic modifications do not alter the sequence of DNA, and they comprise distinct mechanisms such as DNA methylation (DNAme), histone modifications and micro-RNA [26]. Given the scope of this review, we focus our efforts on describing the contribution of DNAme in MS.

## DNA methylation

DNAme is the most current epigenetic hallmark in human somatic cells [27]. The methylation of DNA occurs when a methyl group is transferred to the fifth carbon of a cytosine (5mC) through the action of DNA methyltransferases (DNMT). This process occurs mainly in CG dinucleotides, which are commonly found in the regulatory and promoter regions [28]. The addition of methyl groups in the promoter region contributes to gene silencing [28]. In mammals, DNMTs are mainly represented by DNMT1, DNMT3a and DNMT3b. DNMT1 acts during the cell cycle to maintain the DNAme pattern [29] and participates in the DNA repair system [30]. By contrast, DNMT3a and DNMT3b catalyse the de novo addition of a methyl group into a naked cytosine [31]. This fact occurs in cooperation with either the specific transcription factors or the binding transcription factors, which methylate all the CpG sites uncovered [32].

**Fig. 2:**
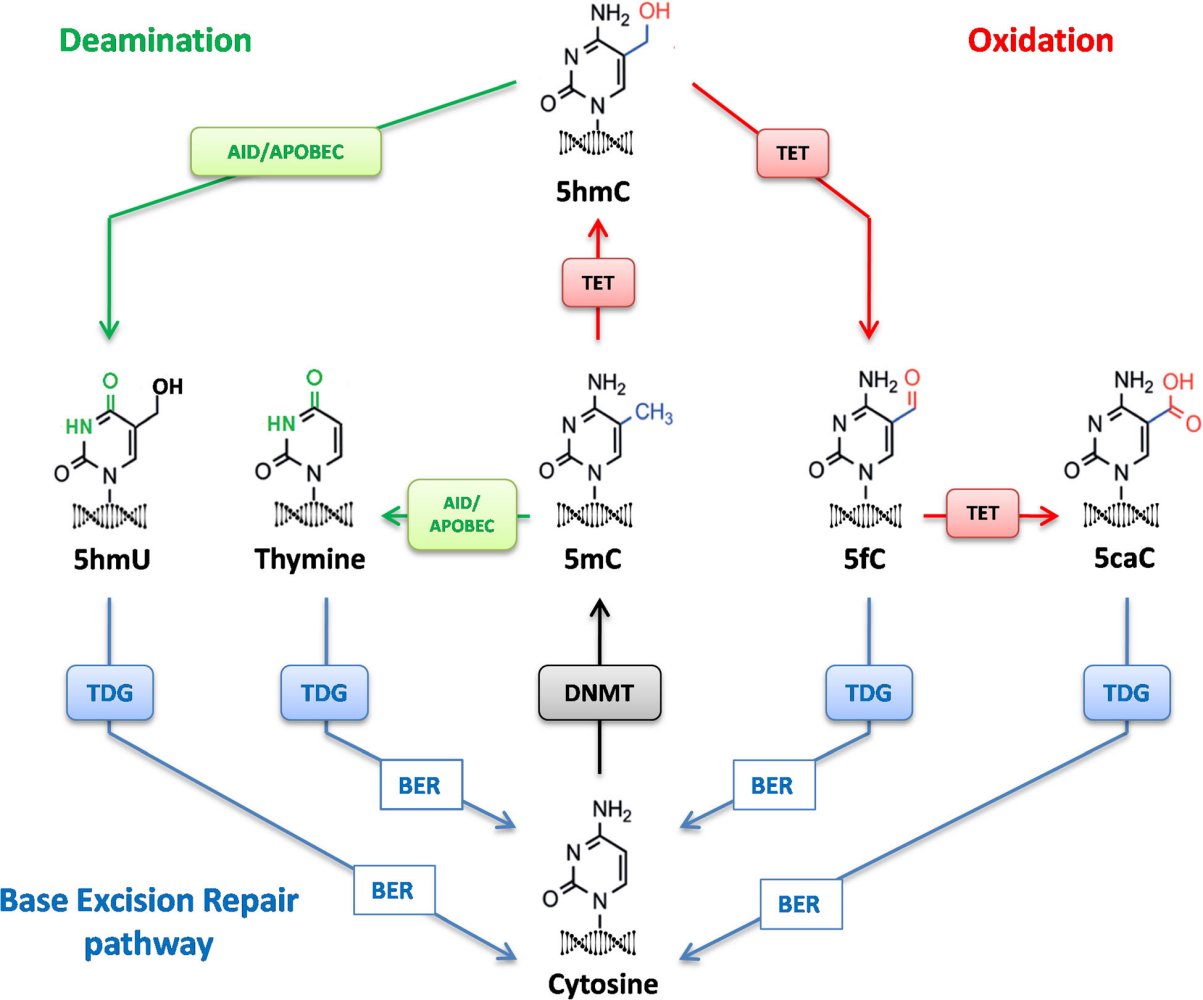
DNA methylation metabolism. The addition of a methyl group to a naked cytosine is catalysed by DNMT (black arrow). 5-methylcytosine (5mC) is oxidised by TET enzymes to 5-hydroxymethylcytosine (5hmC) which can be further oxidised to 5-formylcytosine (5fC) and 5-carboxylcytosine (5caC) as indicated red arrows. In the deamination pathway (green arrows), AID or APOBEC can deaminate 5hmC to 5-hydroxymethyluracil (5hmU) or 5mC to thymine. Eventually, all these modified bases (5hmU, Thymine, 5fC, 5caC) are recognised by TDG and converted to naked cytosine through the base excision repair (BER) pathway (blue arrows)

DNAme is a dynamic process that usually requires the removal of the methyl group (demethylation) to cope with environmental stimuli [33]. This process can be achieved in a passive or active manner. Passive DNA demethylation occurs when DNMT1 activity is misregulated and cannot maintain the integrity of the DNAme pattern during the DNA replication, leading to the incorporation of unmethylated cytosine into the genome [34]. Active DNA demethylation is achieved when a sequence of enzymatic reactions of oxidation and/or deamination modifies 5-methylcytosine (5mC) to obtain a naked cytosine. In the oxidation pathway, 5mC is oxidised by the ten–eleven translocation (TET) enzymes to 5-hydroxymethylcytosine (5hmC) [35], which can be further oxidised to 5-formylcytosine (5fC) and 5-carboxylcytosine (5caC) [36]. In the deamination pathway, AID or APOBEC deaminates 5hmC to 5-hydroxymethyluracil (5hmU) or 5mC to thymine [37]. Eventually, all these modified bases (5hmU, Thymine, 5fC, 5caC) can be recognised by thymine DNA glycosylase (TDG) [38] and converted to naked cytosine through the base excision repair pathway (Fig. 2).

### Contribution of DNA methylation in patients with MS

Although the precise role of the DNAme in MS remains to be fully elucidated, several studies have reported differentially methylated regions in both the immune cells and brain tissue collected from MS patients (Table 1). Therefore, we summarise the current published research on DNAme as follows: BBB breakdown, inflammation, demyelination, remyelination failure and neurodegeneration.

**Table 1:** DNA methylation changes in MS

References	Comparison	Sample target	Method	Differentially methylated genes
[39]	MS vs CTR	CD8+ T cells	Illumina 450K array	ERG, FTL, DCAF4, NCAPH2, CDKN1C, ZNF462, CBX7, MIR492, HPS1, SASH1, MYL3, KCNG1, DYDC2, MEGF10, SP5, LMO3, SLC12A7, MORN1, IGF2BP1, PLCB3, ABCC4, CREG2, CDC42BPB, UGT1A10, TMEM125, ARHGAP22, DACH1, OR8B12, TMEM8C, BAI1, EIF2S1, CRTAC1, DHX36, C19orf41, DLGAP2, TNXB, PRDM8, HEATR2, WHSC2, CAMTA1, ALK, KCNQ2, SCTR, RHEB, LOC202181, RRP9, KRT75, DGKE, PLD5, ZC3H14.
[40]	RRMS vs CTR	CD4+ T cells	Illumina 450K array	MICA, MICB, HLA-DRB, MORN1, LCLAT1, PDCD1, MUC4, AHRR, ARSB, PCBD2, TGFBI, PCDHB13, PCDHB15, KIF25, CSGALNACT1, ADARB2, LDHAL6A, CORO1B, USP35, FUT4, ERC1, TCRA, PACS2, IL32, KCTD11, C17orf108, ARHGAP27, NPLOC4, SBNO2, GNG7, C21orf56, RIBC2.
[41]	Myelinated vs demyelinated MS brains	Hippocampus	Illumina 450K array	MLLT4, PPIF, SCRT2, SNRNP40, ISLR2, MEF2A, PMEPA1, ABCA4, ADAMTS12, AHRR, BEST3, CASP7, CCL4L2, CPXM2, FBXW8, HLA-B, LOC145845, MEIS1, MGMT, MYO7A, NXN, PKP2, PQLC1, PSD3, SCN4B, SDK2, SMYD3, TGFBI, TMEM165, PON1, HDLBP, MKKS, TRIM26, TRPS1, KRTAP27-1, MGP, AJAP1, C1orf106, C2orf62, DSE, EIF2C2, GATA5, HLA-B, IGSF9B, INSC, KIAA1026, KIF25, LOC100292680, NFASC, RASA3, SDK1, SHISA2, SOLH, SORBS2, TAGLN3, TBX5, TM9SF1, TOP1MT, ZSCAN1, AKNA, EBPL, FLJ42709, HERC6, OR52M1, SFRP1, C22orf43, LOC285830, NAPEPLD, NHLH2, PLCH1, SERPINA9, SLFN13, TMEM132B, TTLL3, WDR81.
[42]	SPMS vs CTR	PMBCs	Microarray dataset and RT-PCR	DNMT3A, GADD45A, GADD45B, MBD4, APOBEC3D, APOBEC4, GADD45G, TET1, TDG, APOBEC3C, APOBEC2, MBD2, MBD3, APOBEC3A, DNMT3B, APOBEC1, TET2, TET3.
[43]	RRMS vs PPMS vs CTR	PMBCs	Illumina 450K array	*RRMS vs CTR*: ASB2, ATP11A, CACNA2D3, CERS5, ESRRG, FRMD4A, GNAS, HOXC4-HOXC6, IFITM5, ILDR1, KCNK15, KLHL35, LEFTY2, PLEKHA2, RPH3AL, WRAP73, ZFYVE28.*PPMS vs CTR*: ATG16L2, CES1, CSGALNACT2, CYB5D1, LSMD1, FAM110A, GDF7, HKR1, HLA-F, HOXB13, IGSF9B, ILDR1, LDB2, MTPN, LUZP6, NTN1, OPCML, OR2L13, RBM46, TBX1, TCP10L, TMEM44, VIPR2, WRAP73. *RRMS vs SPMS*: ABCC5, AKAP12, CARS, CBFA2T3, CCDC67, FAM110A, FRMD4A, GIMAP5, HIVEP3, ICAM5, KCNQ1, KLF4, LEFTY2, OLFM3, PTH1R, RASA3, RNF39, RPH3AL, TRAF3, USP35, XKR5.
[44]	Smoker vs non-smoker MS	PMBCs	Bisulphite Illumina Methylation 450k Beadchip	SRM, GNG12, GFI1, ANXA4, NFE2L2, ABLIM2, AHRR, SMIM3, CDKN1A, TPST1, CNTNAP2, SNTG1, MTSS1, PTK2, ZC3H3, ZMIZ1, PTGDR2, PRSS23, GRIK4, ETV6, RARG, LOC348021, CCDC88C, ITPK1, ANPEP, RARA, SMIM6, RECQL5, F2RL3, LINC00111, ACOT9.
[45]	MS vs CTR	NAWM	Direct BS-sequencing	PAD2
[46]	RRMS vs CTR	cfDNA (whole blood)	BS-PCR sequencing assay	MBP3, WM1.
[47]	MS vs CTR	NAWM	Bisulphite Illumina Methylation 450k Beadchip	ALDOA, ATP1A2, BCAR1, BRK1, CDK5, CORO1A, CSF3, DLC1, DTNBP1, FGD2, FMNL1, MLST8, MYBPC3, MYH6, MYH7, MYO1F, OBSCN, PDGFA, PRKCZ, SHC1, SIPA1L1, SSH3, TPM3, ADA, AGAP1, ALDOA, ARHGEF16, ATP1A1, ATP1A2, ATP1A4, ATP5H, BIN1, DAB2IP, DLC1, FGD2, LDHC, MACROD1, MLST8, MYBPC3, MYH6, MYH7, NME4, NT5C, PLXNB1, PTPRN2, RASA3, SEPT9, SIPA1L1, TBCD, TK1, ACSBG1, ACSL1, ACTR8, ADA, AGAP1, AGPAT1, AGRP, AKAP8, ALDH3A1, ALDOA, AMH, ANGPT2, APBB1IP, APEX, ARHGEF16, ATF6B, ATP11A, ATP1A1, ATP1A2, ATP1A4, ATP6V0E1, ATRIP, BBS2, BCAR1, BCL2L2, BIN1, BIRC5, BPI, BRD4, BRK1, C4B, CACNA1D, CASKIN1, CBX4, CCL17, CCL22, CD37, CD59, CDH1, CDK5, CHST3, CHURC1, CLASP1, CLIC5, CORO1A, CREB5, CRY2, CSF3, CSNK1E, CX3CL1, CXXC5, CYP21A2, DAB2IP, DAND5, DCPS, DHRS3, DLC1, DLL1, DOK4, DOT1L, DSCAML1, DTNBP1, DYRK1B, E2F6, E4F1, EDN2, EFS, ENTPD2, ERCC3, F7, FAM109A, FGD2, FGFR3, FMNL1, GBX1, GDF10, GPR114, GPR56, GTF2H1, GYLTL1B, HDAC11, HEG1, HEXIM1, HEXIM2, HIGD1A, HIST3H3, HLA-DMA, IL17RB, IL25, IL34, INO80E, INPP5J, INTS1, IRAK2, ITPKB, JARID2, LIMD1, LMF1, LPCAT1, MAB21L2, MADD, MAML3, MAP3K14, MAPK3, MBP, MCF2L, MED24, MEIS2, MLLT10, MLST8, MT1A, MT1E, MT1F, MT1G, MT1M, MT2A, MT4, MTCH1, MTSS1L, MUSK, MYBPC3, MYH6, MYH7, MYO1F, NARFL, NCOR2, NDRG1, NLRP3, NOTCH4, NR1H3, NUP210, OBSCN, OTX2, PABPN1, PAG1, PBX2, PCSK6, PDGFA, PEG10, PHF21A, PIK3R1, PLEKHG3, PLLP, PLXNB1, POLD4, POLR2C, POU2F1, PPARA, PPIL2, PPP1R13B, PPP4C, PRAM1, PRDM16, PRKCH, PRKCZ, PTGDS, PTPRN2, RAD9A, RAI1, RASA3, RBP1, RFX5, RIN2, RNF187, RPA1, RRM2, RXRA, SACS, SEMA4C, SETD1A, SHC1, SHISA5, SIPA1L1, SLC17A7, SLC22A17, SLC39A13, SLC7A8, SMAD6, SOX1, SOX8, SPI1, SPOCK2, SREBF1, SSH3, SSTR5, SUN1, TACC3, TBCD, TBX6, TEAD2, TEF, TEP1, THRA, TLN2, TNRC6C, TPM3, TRAF2, TSNARE1, UBE2L3, USP19, VAC14, WHSC1, WISP1, WISP2, WNK2, ZBTB47, ZFP1, ZIC1, ZNF135, ZNF256, ZNF329, ZNF362, ZNF414, ZNF418, ZNF488, ZNF606, ZNF664, ZNF687, ADAMDEC1, AIF1, AIRE, B2M, BPI, C1QA, C1QB, C1QC, C4BPA, C4BPB, CCR6, CD19, CD37, CD4, CD7, CD81, CFD, DLG1, FCER2, HAMP, HLA-DMA, HLA-DMB, HLA-DOA, HLA-DOB, HLA-DQA2, HLA-DQB2, HLA-F, IRF6, IRF8, IRF9, JAK1, JAK3, KYNU, LAG3, LAT, LBP, LCP2, LGMN, LST1, LTA, LTB, MBL2, MICB, NCR3, OSM, PSMB8, PTPN22, RARA, RNF31, SECTM1, SLAMF7, STXBP2, TAP1, TAP2, TAPBP, TNF, TNIP2, B2M, C1QA, C1QB, C1QC, C4BPA, C4BPB, DLG1, FCER2, HLA-DMA, LAG3, LTA, MBL2, NCR3, SLAMF7, TAP1, TAP2, TNF, B2M, C1QA, C1QB, C1QC, C4BPA, C4BPB, DLG1, FCER2, HLA-DMA, LAG3, LAT, LTA, MBL2, NCR3, SLAMF7, STXBP2, TAP1, TAP2, TNF, B2M, FCER2, HAMP, LAG3, MBL2, NCR3, SLAMF7, STXBP2, TAP1, TAP2, BHLHE23, CTSZ, DLG1, DLL1, DLX1, DLX2, EDARADD, EPHB4, FOXL2, GLI1, GNAS, HOXC11, HOXC13, HOXC4, HOXC8, HOXC9, HOXD10, HOXD11, HOXD13, HOXD3, HOXD4, HOXD8, HOXD9, MSX1, PHLDA2, PPP1R13L, PTCD2, RARA, RUNX3, SOX1, SOX8, TBX3, TEAD2, TGM1, TH, TNF, TWIST1, WNT2, ZIC1.
[48]	RRMS and CTR	CD4+ T cells CD8+ T cells Whole blood	Illumina 450K array	*CD4+T cells:* DCX, RDH13, DNHD1, TEKT5, TXNL1, MAGI2, TTC30B, APC2, TMEM48, ANGPTL2, RALGPS1, USP29, C20orf151, DLL1 6, DACH2, INPP5A, LOC727677, SEMA5B, SUGT1L1, HOXB2, OR10J5, RBMS1, C20orf151, AEN. *CD8+T cells:* APC2, HOXA2, HRNBP3, HEXDC, NTRK3, DCX, TRIL, ARHGEF17, ESPNP, LHX5, TEKT5, LRRC43, CYP27C1, TMEM48, HHATL, AMMECR1, C19orf45, SRRM3, PSD3, PTPRN2, LOC654342, ARHGEF17, DNHD1, KIF1C, INCA1, VSIG1. *Whole blood:* DACH2, LAMA2, TTLL8, GALNT9, POU3F4, NLRP12, PLS3, ANKRD1, CLSTN2, MAGEB4, APC2, PCDHA7, TMEM27, DNHD1, LGI1, PTCHD2, MMD2, HHATL, TMEM48, NXPH1, TDRD9, CDX1, YTHDC2, RGPD1, PLGLB2.
[49]	RRMS vs PPMS vs SPMS vs CTR	Buffy coat	BS-sequencing	SHP-1
[50]	MS vs CTR	Whole blood PBMCs NAWM	Illumina 450K array	IL2RA
[51]	MS treatment-naïve vs 1 year IFN-b vs CTR	PMBCs	BS-PCR sequencing assay	LINE-1
[52]	Discordant twins (RRMS vs CTR)	CD4+ T cells	RRBS	TMEM1, PEX14.
[53]	RRMS(e)vs RRMS(r) vs CTR	Serum	BS-PCR sequencing assay	MOG
[54]	RRMS vs CTR	cfDNA (serum)	BS-PCR sequencing assay	LINE-1
[55]	RRMS vs CTR	CD3+ T cells	BS-PCR sequencing assay	VDR
[56]	RRMS(e) vs RRMS(r) vs CTR	cfDNA (plasma)	MethDet-56 microarray based assay	*RRMS(r) vs CTR:* CDH1, CDKN2A, CDKN2B, FAS, ICAM1, MCJ, MDGI, MUC2, MYF3, PAX5, PGK1, RB1, SOCS1, SYK, TP73. *RRMS(e) vs CTR:*BRCA1, CCND2, DAPK, FAS, FHIT, MCT1, MDGI, MCJ, CDKN2A, TP73, PGK1, PR-PROX.*RRMS(r) vs RRMS(e):* CDH1, CDKN2B, HIC1, PR-PROX, SYK.
[57]	RRMS(e) vs RRMS(r) vs CTR	Whole Blood	Methylation-Specific Multiple Ligation Probe Amplification PCR	CDKN2A, SOCS1, RUNX3, NEUROG1.
[58]	Discordant twins (MS vs CTR)	PMBCs CD4+ T cells	Bisulphite Illumina Methylation 450k Beadchip	TMEM232, SEMA3C, YWHAGI, ZBTB16, MRI1.
[59]	RRMS and SPMS vs CTR	PMBCs	BS-PCR sequencing assay	PAD2
[60]	RRMS and SPMS vs CTR	PMBCs	EpiTyper assay	DNMT1, TET2
[61]	RRMS vs SPMS vs CTR	CD4+ T cells	Illumina 450K array	VMP1, MIR21

*MS* multiple sclerosis, *CTR* control, *RRMS* relapsing–remitting multiple sclerosis, *PPMS* primary progressive multiple sclerosis, *SPMS* secondary progressive multiple sclerosis, *RRMS(e)* RRMS in exacerbation, *RRMS(r)* RRMS in remission, *cfDNA* circulating-free DNA, *PBMCs* peripheral blood mononuclear cells, *BS* bisulphite, *RRBS* reduced representation bisulphite sequencing, *NAWM* normal appearing white matter

#### BBB breakdown

Infiltration of the autoreactive proinflammatory cells across the BBB into the brain is one of the pathological features of MS [62]. The BBB is a selective semi-permeable endothelium that separates the CNS from the circulating blood. This barrier is composed of a monolayer of endothelial cells tightly bound mainly by cadherins [63] and intercellular adhesion molecule (ICAM) proteins [64]. Cadherins are calcium-dependent adhesion molecules importantly involved in cell–cell adhesion [63]. The disruption of cell–cell interaction mediated by cadherins leads to BBB permeability [63]. A hypermethylated pattern of E-cadherin (CDH1) may increase the BBB permeability in relapsing–remitting MS (RRMS) patients favouring lymphocyte infiltration into the brain, and lastly, disease progression [47, 56]. The other adhesion molecules expressing on the BBB endothelium are the ICAM family. In particular, ICAM-1 is essential for leukocyte crawling prior to diapedesis from the bloodstream to the CNS [65] and plays a remarkable role in T cell proliferation [66]. Liggett et al. (2010) reported a hypermethylation pattern for ICAM1 in cell-free plasma DNA derived from RRMS patients in response to clinical remission, indicating an impairment of the T cell extravasation into the brain as a consequence of immune response mitigation [56]. These findings are in accordance with the results reported in knockout mice for Icam1 subjected to the experimental autoimmune/allergic encephalomyelitis (EAE) model [66].

#### Inflammation

The first inflammatory event in MS is conducted when APC through the class II MHC complex presents a specific antigen to naïve CD4+ T cells, which favour T cell differentiation and the recruitment of proinflammatory cells into the CNS [5]. MHC, also known as human leukocyte antigen (HLA), is responsible for presenting non-self-antigens to the T cell receptors and natural killer receptors (NKRs) [67] facilitating the inflammatory response. Leukocytes use the HLA complex to distinguish self-proteins from exogenous components [68]. In MS, certain HLA genes showed an aberrant methylation pattern contributing to MS aetiology [47]. For example, the hypomethylation of MHC class I polypeptide-related sequence B (MICB) has been reported in normal appearing white matter (NAWM) [47] and CD4+ T cells in MS patients [40]. In MS, a ligand codified by MICB activates the NK and CD8+ T cell destruction [69]. Similarly, the HLA-F variant is actively expressed in the inflammatory reaction [67] as a result of its promoter demethylation [43, 47]. Aside from the HLA complex, changes in DNAme are found in other inflammatory pathways reported in MS. Specifically, global CG island hypermethylation of the Src homology region 2 domain-containing phosphatase-1 and the suppressor of cytokine signalling 1 might aggravate the course of MS through the overactivation of the immune-mediated response [49, 56, 57]. Adhesion molecules such as ICAM5 are markedly present in the cerebral and hippocampal neurons [70]. In MS, the extracellular domain of ICAM5 is cleaved and released into the cerebrospinal fluid (CSF) and blood, where it modulates the synthesis of proinflammatory cytokines (TNF-α, IL-1β), stimulates the expression of anti-inflammatory cytokine IL-10 and represses T cell activation [71]. As a result of disease progression, primary progressive MS (PPMS) patients with non-recoverable demyelination and neurodegeneration showed higher methylation levels for ICAM5 than RRMS patients [43]. This result suggests that an overactivation of the inflammatory response in MS may be attributable to the aberrant methylation pattern of certain anti-inflammatory genes.

#### Demyelination

In MS, demyelination occurs when the myelin sheath of neurons is damaged by the action of the immune system [72]. The attack of immune surveillance is mainly directed against the myelin basic protein (MBP) [73], a protein that stabilises and maintains the correct structure of the myelin sheath around the axon [74]. An extensive hippocampal demyelination simultaneously coincides with the lower number of methyl groups to the DNMT promoters with an increase in their mRNA levels and a decrease in their TET enzymes [41]. Under normal conditions, approximately 20% of the total MBP is citrullinated (MBP-Cit). Citrullination is a post-translational modification catalysed by peptidyl arginine deiminase 2 (PAD2) [75]. The addition of citrullin groups leads to the loss of myelin compaction [76], and particularly, the percentage of MBP-Cit increases drastically [77] along with the promoter demethylation and overexpression of PAD2 as a result of the clinical course [45, 59]. The processing of MBP self-antigens and their presentation by APCs to T cells occurs during the negative selection of autoreactive T cells in the thymus [78]. An increase in the legumain (LGMN) activity, an enzyme involved in the self-antigen processing, prevents the development of immune tolerance against MBP [79]. Interestingly, the demethylation of the LGMN promoter could be responsible for favouring autoimmunity in MS patients [47, 74].

#### Remyelination failure

Following the myelin destruction, the recruitment of oligodendrocyte progenitor cells (OPCs) is necessary to rescue the demyelinated axons [80]. However, in MS patients, this process is not completely achieved [81], contributing to progressive neurodegeneration. The origin of this failure is not fully understood, but some hypotheses have been postulated in this regard [81]. Briefly, remyelination may be incomplete because of the inadequate recruitment of OPCs into the demyelinated lesion, an impairment of the OPC differentiation into myelinating oligodendrocytes, or the dysfunctions in oligodendrocytes when they attempt to wrap axons [81, 82]. In adults, OPC migration and recruitment require several growth factors including the platelet-derived growth factor (PDGF) and the fibroblast growth factor (FGF) [83–85]. Both growth factors are significantly methylated in the NAWM of MS patients [47]. In this regard, the addition of methyl groups to the DNA may be accompanied by the lower expression of PDGF and FGF during disease progression, thus gaining mechanistic insight into the oligodendrocyte dysfunction. Wnt signalling pathway is involved in the differentiation of precursor oligodendocytes into mature myelinating oligodendocytes, affecting remyelination of axons [86]. Fancy et al. (2009) found that MS demyelinating lesions display an activation of the Wnt signalling impairing the remyelination process due to lack of proliferation of premyelinating oligodendrocytes [86]. Interestingly, some of the negative regulators of the Wnt pathway (histone deacetylase inhibitors, or Shisha genes) [87, 88] are hypermethylated in the brain [47, 41]. This hypermethylation would facilitate Wnt pathway activation declining efficiency of endogenous remyelination. This hypothesis is reinforced by Chen et al. (2011). They reported that mice lacking HDAC1/2 displayed severe myelin deficiency [89]. The hypermethylation of neurofascin (NFAS) has been related to the improper myelin wrapping of paranodal segments and might respond to the pathophysiological mechanisms underlying MS [90, 91]. Self-antibodies against NFAS have been detected in MS patients [92]. In the hippocampus while neurodegeneration is ongoing, Chomyk et al. (2017) reported a decrease in the WD repeat domain 81 and AT-hook transcription factor methylation status, which could lead to a lower mRNA expression and neuronal injury [41].

#### Neurodegeneration

Progressive axonal loss contributes to brain atrophy and neurological disability [93]. Kulakova et al. (2016) examined the DNAme of the peripheral blood mononuclear cells (PBMCs) collected from secondary progressive (SPMS) and RRMS patients, providing a time course DNAme pattern [43]. They found a cluster of 21 differentially methylated genes. Gene ontology analysis revealed that four of these genes are hypermethylated while neurodegeneration is ongoing and involved in biological processes, such as cell proliferation (PTH1R, CBFA2T3, KLF4) and nuclear factor κ-light-chain-enhancer of activated B cell (NF-kβ) pathway (KLF4, TRAF3). NF-kβ is a pleiotropic regulator of neuroinflammation, neuronal protection and neurotoxicity [94] involved in distinct pathological events mediated by the glia once the earliest signs of MS are present [95]. During the course of neurodegeneration, an imbalance between the expression of enzymes participating in the addition and removal of methyl groups to the DNA has been reported in MS patients. Indeed, Fagone et al. (2016) found an upregulation of methyl CpG-binding protein 2 and 4 and a downregulation of enzymes such as TET3 and TDG in SPMS patients [92].

## Environmental and lifestyle risk factors

**Fig. 3:**
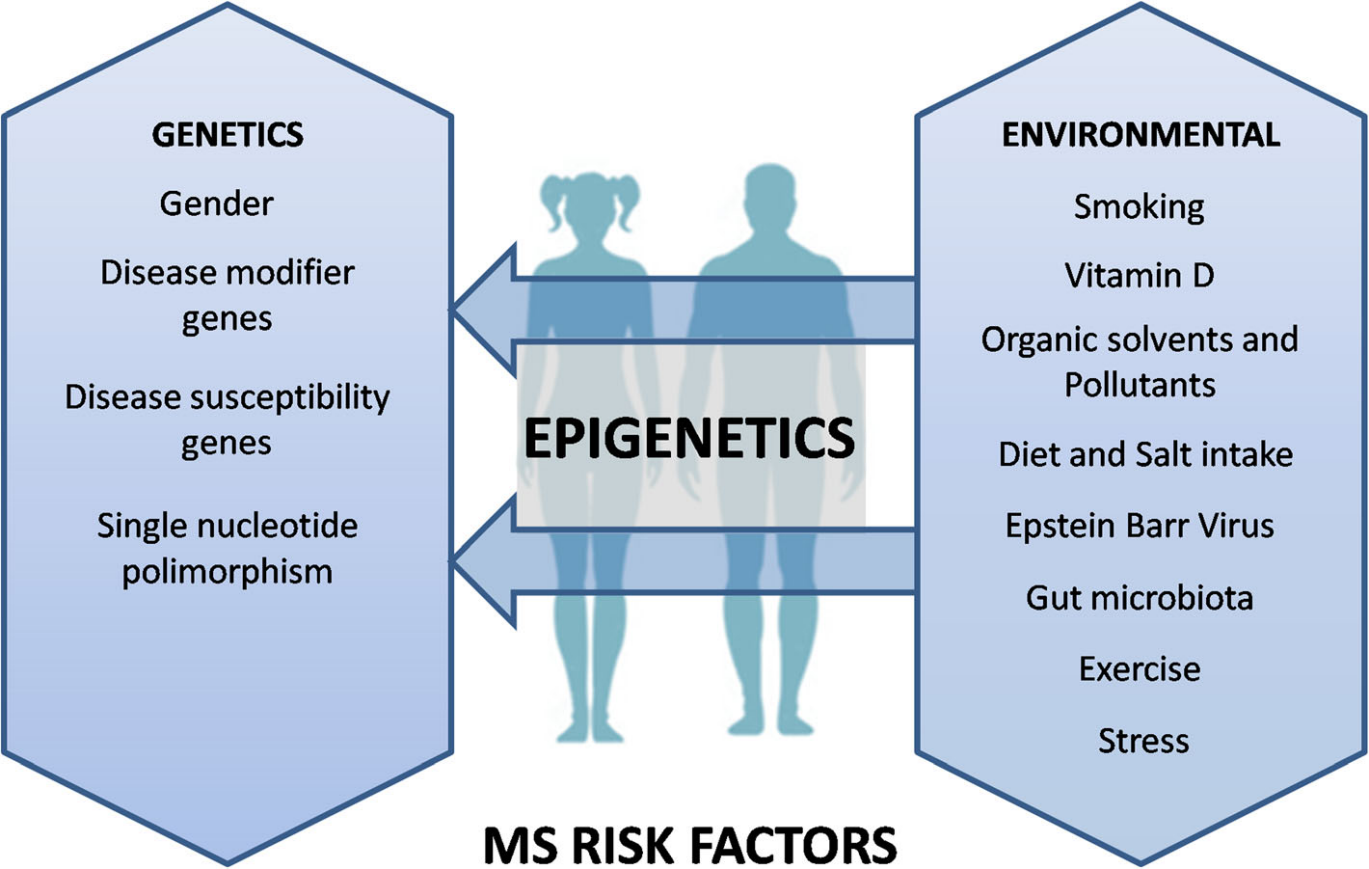
Risk factors of multiple sclerosis. MS pathogenesis is influenced by both genetic and environmental factors. Among the genetic factors, gender, disease-modifier genes, disease susceptibility genes and single nucleotide polymorphisms are remarkably important in prevalence and MS pathogenesis. In contrast, environmental factors such as smoking, vitamin D deficiency, organic solvents and pollutants exposure, diet style, Epstein Barr infection, dysbiosis of the gut microbiota, lack of exercise and stress are critically associated with MS susceptibility and progression

The influence of the environment on MS pathophysiology has been largely studied in twins discordant for the disease [52]. This divergence has been attributed to the effect of environmental risk factors through epigenetic modifications [96]. Thus, Handel et al. (2010) reported an additive deleterious effect of smoking, ultraviolet (UV) exposure and HLA alleles on MS prevalence [20]. Scientific evidence shows that environmental factors, such as smoking, Epstein Barr infection, vitamin D level, organic solvent and chemical pollutant exposure, diet style, gut microbiota, exercise and stress, are involved in the development and/or course of MS (Fig. 3).

### Smoking

Smoking confers the risk for developing MS, and it is associated with disease onset and progression [97]. Degelman and Herman (2017) found a significant association between smoking frequency and conversion from RRMS to SPMS forms [98]. Nearly 98 chemical compounds of tobacco, including nicotine, cyanide and nitric oxide, are hazardous [99]. For example, nicotine increases the permeability of the BBB [100] in the earlier stages of MS [101], cyanide contributes to CNS demyelination [102] and nitric oxide promotes neurodegeneration [103]. Tobacco contains dioxins that activate the aryl-hydrocarbon receptor pathway [104], which modulates neuroinflammation [105] and Th17 and Treg activities [106] becoming a key player in the MS aetiology and disease progression. A well-designed study performed by Zeilinger et al. (2013) reported the differences in the DNAme of current, former and never smokers [107]. They found that the aryl-hydrocarbon receptor repressor (AHRR) was highly demethylated among the current smokers, leading to the inhibition of AhR signalling pathway and thus enhancing neuroinflammatory and neurodegeneration events [105, 106]. AhR can act as protective or deleterious pathway depending on the cell type where is expressed. In EAE model, AhR activation has a protective role in dendritic cells (DC), astrocytes and foxp3+ T reg cells, while it has a pro-inflammatory effect in Th17 cells [108]. AhR deletion is known to exacerbate EAE disease [109], altering myelin-associated proteins and increasing the production of proinflammatory cytokines [110]. In MS patients, low levels of AhR have been measured in serum, along with a reduced AhR activity in demyelinating lesions during disease progression [111]. Interestingly, Laquinimod, a phase III drug for MS treatment that activated AhR pathway, was shown to reduce brain atrophy in MS patients by counteracting the neuroinflammatory reaction [112].

### Epstein Barr virus

The latent form of EBV is present in almost 90% of the world population within memory B cells [113]; more than 99% of MS patients are seropositive for EBV [24]. Infection with EBV increases the risk of developing MS by ~ 3.6-fold [114], and its effect can be exacerbated when interacts with other risk factor, such as HLA risk variants, rocketing the odd ratio for MS up to ~ 15-fold [115]. We can speculate that a cross-reaction may occur between the myelin self-antigens and certain viral proteins of the EBV in MS [116]. This hypothesis is underpinned by the identification of two EBV peptides (EBNA-1 and BRRF2) in the CSF of MS patients [117]. Indeed, the CD8+ T cells derived from MS patients displayed reactivity against some latent EBV proteins [117]. The reactivation of latent EBV in memory B cells may occur through specific epigenetic modifications. For example, the use of 5-azacytidine, a DNMT inhibitor, can switch the EBV latent form to a reactivated form [118].

### Vitamin D

Vitamin D is synthesised upon exposure to UV [119] or can be obtained from diet [120]. Vitamin D deficiency is considered a risk factor for MS even before birth, altering the structure of embryonic tissues as a result of low maternal vitamin D [121]. Indeed, the prevalence of MS is lower near the equator, where UV radiation is at its maximum, than at higher and lower latitudes [122]. Low levels of vitamin D have been associated with a higher frequency of relapses [123] and disability [121].

Current evidence points out that the active form of vitamin D acts as an inducer of the promoter demethylation of multiple genes [124]. In accordance with these findings, Rawson et al. (2012) reported that a high intake of vitamin D is associated with lower methylation levels of certain genes involved in the Wnt signalling pathway [125], which could favour neurogenesis and neuronal plasticity [126]. In T cells from RRMS whole peripheral blood, the vitamin V receptor (VDR) showed a promoter demethylation pattern associated with an overexpression of VDR mRNA [55]. Nevertheless, the underlying mechanism behind vitamin D and its effect on demethylation remains unknown and requires further investigation.

### Organic solvents and pesticides

Organic solvents are hydrocarbon compounds commonly used worldwide. The prolonged exposure to these compounds has severe effects on health and may be clinically important in autoimmune diseases [127]. Organic solvents are highly hydrophilic and lipophilic molecules able to pass through the BBB into the brain, resulting in distinct myelin pathologies [128]. Exposure to organic solvents induces changes in the DNAme of the immune system [129] and certain genes involved in cell survival [130]. Hexachlorobenzene, a pesticide widely used until 1965, modulates the expression of E-cadherin through the activation of the integrin-linked kinase signalling [131]. E-cadherin plays an important role in BBB integrity, and its promoter region is highly methylated in MS patients [49], facilitating the infiltration of immune cell into the brain. To evaluate the precise contribution of this pesticide in the context of MS, we should determine the exposure time frame and if it occurred before or after the diagnosis of disease. Remarkably, the precise effect of organic solvents on DNAme in MS warrants further investigation. However, exposure to organic solvents and the presence of HLA risk alleles present a fourfold increased risk for MS in comparison with exposure to organic solvents alone, and the additive effect of organic solvents, HLA risk alleles and smoking increase the risk 20-fold [132].

### Diet

Recent evidence points out that food intake is important in the pathogenesis of MS and other autoimmune diseases. Seasonal variations in food intake in pregnant mothers may interfere with foetal development and confer the risk of MS in the later life of the offspring (for a review, see [133]). The intake of some nutrients, such as long-chain fatty acids and salt, is known to act as pro-inflammatory molecules, whereas other nutrients, such as short-chain fatty acids and flavonoids, possess anti-inflammatory properties [134]. A type of flavonoid called quercetin represses the capacity of monocytes to cross the BBB [135] and reduces the synthesis of proinflammatory cytokines produced by monocytes in MS [136]. Limited data are available on the intake of nutrients and their effect on DNAme. However, certain cofactors obtained from diet are required to maintain DNAme homeostasis. For example, vitamin B, folate, methionine, choline and zinc are essential to maintain the levels of Dnmt1 [137] and methyl-donor S-adenosylmethionine [137]. By contrast, vitamin C can act as a cofactor for TET enzymes [138]. In particular, vitamin B_12_ is necessary for the proper function of the CNS through the conversion of homocysteine to methionine. Methionine regulates the expression of DNA methyltransferase 3A (DNMT3A) [139] and participates in the transcription of pro-inflammatory genes and the formation of myelin sheath [140]. Interestingly, vitamin B_12_ and folate deficiency is reported in RRMS concomitantly with elevated levels of homocysteine. A misbalance in DNAme metabolism has been reported in MS patients [139, 141]. Indeed, some authors found that the levels of methyl group donors were lower in plasma [139] and post-mortem grey matter [141] in MS.

The elevated consumption of salt has many noxious effects, including the production of reactive oxygen species [142], deregulation of regulatory T cells [143] and macrophage [144], disruption of BBB permeability [145] and higher probability of new brain lesions in RRMS patients [146]. After the ingestion of a diet containing high levels of salt, patients with an autoimmune disease showed demethylation and elevated levels of hydroxymethylation in CD4+ T cells as a result of TET2 overexpression [25]. Consequently, the high consumption of salt may be a risk factor for MS. Note that the use of standardised diets or alternative therapies cannot substitute conventional MS treatments, but the intake of healthy food may ameliorate the inflammatory and physical status of MS patients. Therefore, it can be hypothesised that some components of diet may interfere with the DNAme metabolism even before childbirth or during lifetime contributing to both disease aetiology and progression.

### Gut microbiota

Gut microbiota is a complex ecosystem of microorganisms that establish a symbiotic relationship with the host by favouring the vitamin production and fermentation of some components of diet [147]. However, the dysbiosis of microbiota increases the risk of developing autoimmune diseases [148, 149]. DC from germ-free mice subjected to the EAE model were less reactive to stimulated proinflammatory T cells than conventionally colonised germ mice [149]. Berer et al. (2011) found that gut microbiota in cooperation with myelin auto-antigens is necessary to stimulate the immune response [148], consistent with [149]. Dysbiosis has been associated with an inflammatory phenotype in MS patients [150, 151]. Resident microbiota can alter the epigenetic signature of the host through the production of specific metabolites (Table 2). Pregnant women showed a different DNAme profile according to their predominant microbiota in the gut [166]. Therefore, dysbiosis of microbiota can be involved in disease onset by an overactivation of T cells [148, 149] or exacerbating inflammatory events in patients diagnosed with MS [150, 151]. However, the contribution of microbiota in disease aetiology and progression warrants further investigation.

**Table 2:** List of metabolites released by microbiota

Metabolite	Effect on DNA methylation
p-Cresol	It induces the expression of DNA methyltransferases 1, 3a, and 3b and it is associated with CpG hypermethylation of Klotho gene [152], a regulator of vitamin D metabolism [153].
Hydrogen sulphide (H_2_S)	Involved in the neutralisation of ROS. It increases DNA methylation [154].
Riboflavin (vitamin B_2_) Pyridoxine (vitamin B_6_) Cobalamin (vitamin B_12_)	Cofactor involved in DNA methylation metabolism [155, 156].
Folate (vitamin B_9_)	It acts as a methyl donor involved in DNA methylation metabolism [155, 156].
	It reduces the activity of DNA methyltransferase [157].
Choline	It acts as a methyl donor that can be recruited by human gut microbiota, reducing its availability [158].
	Involved in DNA methylation and gene expression in murine colitis model, an inflammatory disease [159].
Betaine	It acts as a methyl donor involved in DNA methylation reactions [156, 160].
	Associated with changes in DNA methyltransferases and coupled with changes in DNA methylation [161].
Ammonium (NH_4_)	Inverse correlation between faecal NH_3_ and LINE-1 gene methylation [162].
*α-*ketoglutarate	Involved in (de)methylation as a co-factor of histone demethylases and TET family [163, 164].
L-ascorbic acid (vitamin C)	It exerts a strong influence on active DNA demethylation. It enhances TET-mediated generation of 5-hydroxymethylation [165].

*ROS* reactive oxygen species, *NH*_*3*_ ammonia, *LINE-1* long interspersed element-1, *TET* ten–eleven translocation

Adapted from Mischke et al. [147]

### Physical activity

Physical exercise has been demonstrated to produce changes in the leukocyte DNAme pattern [167] and thus changes in gene expression [168]. In the context of neurodegenerative diseases, the influence of physical exercise has a direct effect on the brain-derived neurotrophic factor (BDNF) [169, 170]. In a healthy brain, BDNF is mainly expressed in neurons [171]. However, following a demyelinating insult, this gene is transcriptionally active in astrocytes [172], regulatory T cells, B cells and monocytes [172, 173], favouring brain plasticity [174] and myelin formation [175]. Recently, Briken et al. (2016) found elevated protein levels of serum BDNF after 30 min of exercise in progressive MS patients [170], and this result was probably attributable to the demethylation of its promoter region [169]. Similarly, 2 weeks of physical exercise promotes the overexpression of TET1 and the demethylation of the vascular endothelial growth factor A (VEGF-A) [176]. VEGF-A may potentiate neurogenesis and neuroprotection in the EAE model as postulated by [177]. The overexpression of apoptosis-associated, speck-like protein containing a C-terminal caspase recruitment domain gene (ASC) activates inflammatory signalling and may exacerbate MS progression [178]. In addition, Nakajima et al. (2010) found that following 6 months of moderate exercise, the mRNA levels of ASC were lower because its promoter region was hypermethylated [179]. Therefore, the current data support the notion that moderate exercise can reduce pro-inflammatory cytokines and improve the clinical MS course. However, some studies failed to validate this evidence [180–182]. The findings reported here point out that moderate exercise can ameliorate some symptoms but cannot stop the progression of MS.

### Stress

Stressful life events have a negative effect on MS by increasing the risk of clinical exacerbation and disease progression [183]. However, the contribution of stress in the pathophysiology of the disease remains under discussion. A well-designed study by Liu et al. (2009) reported a strong association between stress and MS aetiology [184]. In accordance with these findings, Babenko et al. (2015) demonstrated that prenatal stress causes a demethylation of the nuclear subfamily 3 group C member 1 glucocorticoid receptor (NR3C1) [185] and changes in the nervous, immune and musculoskeletal systems [186]. Interestingly, the differences in NR3C1 gene expression have been reported in MS patients [187], suggesting that early life stressors can present susceptibility to developing MS in adulthood.

## DNA methylation in animal models of MS

None of the current experimental animal models can reproduce the complexity and heterogeneity of MS. In particular, both disease onset and clinical course in animals differ considerably from those in humans [188]. However, in vivo experimental models are widely used to understand certain aspects of the disease. In general, we consider three models to study MS pathophysiology: (a) EAE, (b) Theiler’s murine encephalomyelitis virus (TMEV) and (c) use of toxins such as cuprizone (CPZ) or lysolecithin. However, not all of them have been addressed to study the changes in DNAme. For example, no current study has been conducted on DNAme in the TMEV model.

### EAE model

EAE is a well-established model of autoimmunity induced by the subcutaneous injection of self-antigens derived from myelin proteins, such as the myelin oligodendrocyte glycoprotein (MOG) [189] and the proteolipid protein (PLP) [190]. Catanzaro et al. (2016) characterised the DNAme profile in the striatum of EAE mice showing a global DNA hypomethylation of interneurons positive for neuronal nitric oxide synthase [191]. Interestingly, they found a demethylation of Ras-related protein-1 (Dexras-1) in parallel with elevated levels of iron inside the cells and thus neurotoxicity and neuronal death. Furthermore, they reported that the hypomethylation of Dexras-1 was reverted when mice were subjected to an enriched environment in their home cage, emphasising an epigenetic-mediated effect [191]. Recently, Noori-Zadeh et al. (2017) found that the promoter region for forkhead box P3 was hypermethylated in T cells collected from EAE mice [192], thus indicating a dysfunction of regulatory T lineage and the lack of auto-immune tolerance [193].

### Toxin-induced demyelination

Demyelination can be induced by copper-chelating agents (e.g. CPZ) or lysolecithin [194]. In the CPZ model, young adult mice fed with this neurotoxicant showed a significant loss of mature oligodendrocytes, astrocytosis, microgliosis and demyelination, followed by spontaneous remyelination [195]. The CPZ model was used by Olsen et al. (2019) to identify novel biomarkers related to the demyelination course [53]. Specifically, they isolated circulating-free DNA from mice blood at the end of the CPZ treatment, and identified a specific methylation pattern associated with oligondendrocyte apoptosis. Conversely, the use of lysolecithin in mice induced demyelination accompanied by a high expression of DNMT1 in the OPCs at the early stages of remyelination. By contrast, DNMT3A is highly expressed in oligodendrocytes at the later stages when remyelination is achieved. The study revealed a global hypermethylation in the oligodendrocyte lineage during remyelination, demonstrating that DNMT1 plays a crucial role in the proliferation and differentiation of OPCs into mature oligodendrocytes, while DNMT3A has a dominant role in the remyelination phase [196].

## Conclusions

MS is an inflammatory autoimmune disease of the CNS caused by a complex interaction between genetic and environmental factors [20]. Emerging evidence indicates that DNAme actively participates in gene x environment interactions [33]. As previously mentioned, several studies showed an aberrant DNAme profile in relapsing–remitting forms and in progressive MS forms. Remarkably, most of the studies reported in this work were based on the bisulphite technique (Table 1). However, this approximation does not discriminate between 5-methylcytosine and 5-hydroxymethylcytosine and thus may contribute to a misinterpretation of the data. To avoid this bias, we recommend an alternative method for studying DNAme, such as the methylated-DNA immunoprecipitation and the TET-assisted bisulphate sequencing. As far as we know, genetic factors can explain approximately 30% of worldwide MS prevalence [23], and the remaining 70% may correspond to the influence of environmental risk factors. As described in this study, UV radiation, cigarette smoking and infection with the Epstein Barr virus are clinically relevant for MS, although other environmental factors, such as diet style, microbiota profile, exposure to organic solvents and pollutants, exercise and long-term stress, have a clear effect in MS. All of the aforementioned risk factors can modify the DNAme pattern in humans, but further studies are required to expand our knowledge of the molecular basis of the disease and elucidate the underlying mechanisms behind MS pathophysiology. Furthermore, DNA methylation is currently the best surrogate marker for epigenetic change in disease, because methylation alterations track with disease state. DNA methylation markers can also indicate success or failure of drug treatment, are stable in isolated DNA, and can be measured by a variety of quantitative and qualitative methods. We postulate that epigenetic DNAme marks described in the context of the disease can potentially be used in a specific, substantial, and credible way in clinical interventions. It is conceivable that, in the near future, we will be able to design drugs modifying DNAme metabolism to stop the progression of MS.

## Data Availability

The authors confirm that the data supporting the findings of this study are available within the manuscript.

## Abbreviations

5caC: 5-Carboxylcytosine
5fC: 5-Formylcytosine
5hmC: 5-Hydroxymethylcytosine
5hmU: 5-Hydroxymethyluracil
5mC: 5-Methylcytosine
AHRR: Aryl-hydrocarbon receptor repressor
APC: Antigen presenting cells
ASC: Apoptosis-associated speck-like protein containing a C-terminal caspase recruitment domain
BBB: Blood–brain barrier
BDNF: Brain-derived neurotrophic factor
BER: Base excision repair
BS: Bisulphite
CDH1: E-cadherin
cfDNA: Circulating-free DNA
Cit: Citrullination
CNS: Central nervous system
CPZ: Cuprizone
CSF: Cerebrospinal fluid
CTR: Control
CYP1A1: Cytochrome P450 family 1 subfamily A member 1
DC: Dendritic cell
Dexras-1: Ras-related protein-1
DNAme: DNA methylation
DNMT: DNA methyltransferases
EAE: Experimental autoimmune/allergic encephalomyelitis
EBV: Epstein Barr virus
FGF: Fibroblast growth factor
FOXP3: Forkhead box P3
H_2_S: Hydrogen sulphide
HCB: Hexachlorobenzene
HDACs: Histone deacetylase inhibitors
HLA: Human leukocyte antigen
ICAM: Intercellular adhesion molecules
IFN-γ: Interferon-gamma
ILK: Integrin-linked kinase
LCFA: Long-chain fatty acids
LGMN: Legumain
LINE-1: Long interspersed element-1
MBD: Methyl CpG-binding protein
MBP: Myelin basic protein
MeDIP: Methylated-DNA immunoprecipitation
MHC: Major histocompatibility complex
MICB: MHC class I polypeptide-related sequence B
MOG: Myelin oligodendrocyte glycoprotein
MS: Multiple sclerosis
NaCl: Salt
NAWM: Normal appearing white matter
NFAS: Neurofascin
NF-kβ: Nuclear factor k-light-chain-enhancer of activated B cells
NH_3_: Ammonia
NH_4_: Ammonium
NKRs: Natural killer receptors
nNOS: Neuronal nitric oxide synthase
NR3C1: Nuclear subfamily 3 group C member 1 glucocorticoid receptor
OPCs: Oligodendrocyte progenitor cells
PAD2: Peptidil arginine deiminase 2
PBMCs: Peripheral blood mononuclear cells
PDGF: Platelet-derived growth factor
PLP: Proteolipid protein
PPMS: Primary progressive multiple sclerosis
ROS: Reactive oxygen species
RRBS: Reduced representation bisulphite sequencing
RRMS(r): RRMS in remission
RRMS: Relapsing–remitting multiple sclerosis
RRMS(e): RRMS in exacerbation
SAM: Methyl-donor S-adenosylmethionine
SCFA: Short-chain fatty acids
SHP-1: Src homology region 2 domain-containing phosphatase-1
SOCS1: Suppressor of cytokine signalling 1
SPMS: Secondary progressive multiple sclerosis
TAB-seq: TET-assisted bisulphate sequencing
TCRs: T cell receptors
TDG: Thymine DNA glycosylase
TET: Ten–eleven translocation
Th: T helper
TMEV: Theiler’s murine encephalomyelitis virus
VDR: Vitamin D receptor
VEGF-A: Vascular endothelial growth factor A
Vit D: Vitamin D
